# Modulating DNA damage response in uveal melanoma through embryonic stem cell microenvironment

**DOI:** 10.1186/s12885-024-12290-x

**Published:** 2024-04-24

**Authors:** Yingxu Zhang, Jinbiao Zheng, Minyu Chen, Shulun Zhao, Ruiqian Ma, Wenwei Chen, Jiahui Liu

**Affiliations:** Ophthalmology Department, The Tenth Affiliated Hospital, Southern Medical University (Dongguan People’s Hospital), 78 Wandao Road, Dongguan, 523000 China

**Keywords:** Uveal melanoma, Embryonic stem cells, Nonhomologous end joining, DNA damage response, Microenvironment

## Abstract

**Background:**

Uveal melanoma (UVM) is the most common primary intraocular tumor in adults, with a median survival of 4–5 months following metastasis. DNA damage response (DDR) upregulation in UVM, which could be linked to its frequent activation of the PI3K/AKT pathway, contributes to its treatment resistance. We have reported that embryonic stem cell microenvironments (ESCMe) can revert cancer cells to less aggressive states through downregulation of the PI3K signaling, showing promise in modulating the DDR of UVM.

**Methods:**

Since nonhomologous end joining (NHEJ) is the main DNA repair mechanism in UVM, this study utilized gene expression analysis and survival prognosis analysis to investigate the role of NHEJ-related genes in UVM based on public databases. Xenograft mouse models were established to assess the therapeutic potential of ESC transplantation and exposure to ESC-conditioned medium (ESC-CM) on key DNA repair pathways in UVM. Quantitative PCR and immunohistochemistry were used to analyze NHEJ pathway-related gene expression in UVM and surrounding normal tissues. Apoptosis in UVM tissues was evaluated using the TUNEL assay.

**Results:**

PRKDC, KU70, XRCC5, LIG4 and PARP1 showed significant correlations with UM progression. High expression of PRKDC and XRCC5 predicted poorer overall survival, while low PARP1 and XRCC6 expression predicted better disease-free survival in UVM patients. ESCMe treatment significantly inhibited the NHEJ pathway transcriptionally and translationally and promoted apoptosis in tumor tissues in mice bearing UVM. Furthermore, ESC transplantation enhanced DDR activities in surrounding normal cells, potentially mitigating the side effects of cancer therapy. Notably, direct cell-to-cell contact with ESCs was more effective than their secreted factors in regulating the NHEJ pathway.

**Conclusions:**

Our results suggest that NHEJ-related genes might serve as prognostic markers and therapeutic targets in UVM. These findings support the therapeutic potential of ESC-based therapy in enhancing UVM sensitivity to radiochemotherapy and improving treatment outcomes while minimizing damage to healthy cells.

**Supplementary Information:**

The online version contains supplementary material available at 10.1186/s12885-024-12290-x.

## Background

Uveal melanoma (UVM) is the most common primary intraocular malignancy in adults. As a highly aggressive form of ocular cancer originating in uveal melanocytes, it poses a significant threat to ocular vision and even causes cancer-related death. Although radiation and surgery are effective therapeutic strategies for primary tumors, up to 50% of patients subsequently develop metastasis [1]. UVM is highly susceptible to metastasis to the liver with a median survival of 6–12 months [2, 3]. Despite advancements in treatment modalities, the prognosis for patients with metastatic UVM remains poor because of its treatment resistance, making it one of the few cancers with stable mortality over the past three decades [4].

Radiation or genotoxic chemotherapy exerts lethal effects by causing DNA damage. Activation of the DNA damage response (DDR) in cancer cells can ensue [5]. Recent evidence has begun to suggest a consensus on the upregulation of DDR proteins in UVM [6–8], potentially explaining the well-documented resistance of UVM to radiotherapy and chemotherapy. If DNA repair is incomplete, cancer cells cannot maintain genome integrity and undergo apoptosis and death [9, 10], showing that defective DDR may be an attractive target for UVM treatment.

Studies have shown that the embryonic microenvironment can revert cancer cells into non-cancerous or less aggressive states, such as metastatic melanoma cells and myeloid leukemia cells [11, 12]. These discoveries have garnered attention regarding the embryonic microenvironment in the field of cancer treatment. As embryonic stem cells (ESCs) can provide and maintain a microenvironment similar to the embryonic microenvironment, they have been shown to have suppressive effects on a variety of tumors [13, 14]. Similarly, we have previously established an embryo-like microenvironment using mouse ESCs and indicated that such a microenvironment can effectively induce cell cycle arrest and cell apoptosis of UVM cells both in vitro and in vivo through inhibition of PI3K signaling without damage to normal somatic cells. [15] As inhibition of the PI3K-AKT pathway can downregulate DNA damage repair factors, increasing the sensitivity of cancer cells to radiochemotherapy [16], ESCMe has the potential to modulate the DDR of tumors.

Due to the pivotal role of DDR as a determinant of resistance to therapy in UVM [7–9], inhibition of DNA repair mechanisms can render cancer cells more vulnerable to genotoxic insults, leading to increased DNA damage accumulation and compromised cell survival. This study used bioinformatic methods to detect the expression of the core DDR factors, aiming to screen the genes closely related to the development and prognosis of UVM through clinical big data. Furthermore, we sought to investigate the hypothesis that ESCMe can inhibit the DDR of UVM in vivo using xenograft mouse models. We focused on assessing the impact of ESCMe on key DNA repair pathways in UVM. Additionally, the effects of different ESCMe, including ESCs and ESC-conditioned medium (ESC-CM), on DNA repair modulation were also explored. By elucidating the interactions between ESCMe and DNA repair processes, the findings from this study hold the potential to pave the way for the development of innovative strategies that enhance the efficacy of conventional cancer treatments, such as chemotherapy and radiotherapy.

## Methods

### Gene expression analysis

The UALCAN portal (http://ualcan.path.uab.edu/analysis-prot.html) [14] was utilized to explore transcription in UVM subgroups based on clinicopathological features in the TCGA database. We also collected a publicly available RNA-seq dataset (Accession ID: E-GEOD-22138) [15] from BioStudies [16], and explored the relationship between gene expression and metastasis.

## Survival prognosis analysis

TIMER (https://cistrome.shinyapps.io/timer/), UALCAN, and GEPIA 2 (http://gepia2.cancer-pku.cn/#index) databases can be used to perform survival analysis of specific genes. UVM patients were divided into low and high expression groups based on the values of mRNA expression and validated by survival curves. The methods used were overall survival (OS) and disease-free survival (DFS) with a cutoff of 50%.

## Cell cultures

Human uveal melanoma cell (C918) was obtained from KeyGen Biotechnology Company (China) and cultured in RPMI 1640 medium (Corning, USA) supplemented with 1% penicillin–streptomycin (Gibco, Japan) and 10% fetal bovine serum (FBS; Corning). Mouse ESCs were gifts from Professor Peng Xiang from Sun Yat-sen University. ESCs were cultured by knockout Dulbecco’s modified Eagle’s medium (Gibco) containing 10% FBS, 0.1 mM non-essential amino acid (Gibco), 1% GlutaMAX media (Gibco), 0.055 mM 2-mercaptoethanol (Gibco), 5 × 10^5^ units leukemia inhibitory factor (Millipore, USA), and 1% penicillin–streptomycin.

### Animal experiments

We injected 1 × 10^6^ C918 cells subcutaneously into the right flanks of male Balb/c nude mice as previously described [17]. We collected ESC-CM from cultured ESCs every day and then filtered through a 0.22-mm filter (Millex, USA). ESC-CM was stored at –20 °C. ESCs were collected after culture and resuspended in PBS prior to injection into tumors. Mice were randomized to receive treatment with ESCs, ESC-CM, or phosphate-buffered saline (PBS) when the tumor volume reached 150 mm^3^. ESCs (5 × 10^5^ cells/tumor in 200 μl PBS), ESC-CM (200 μl/tumor), or PBS (200 μl/tumor) was administrated at 2 different sites peritumorally every 7 days. When ESCs are differentiated, their ability to reverse tumor is significantly reduced or completely abolished. ESCs with a suicide gene, herpes simplex virus thymidine kinase (HSV-TK), controlled by ganciclovir (GCV), were constructed previously. GCV (Sigma, 2 mg/mouse in 200 μl PBS) was injected intraperitoneally on day 5 of every treatment cycle to eliminate the differentiated ESCs and avoid the formation of teratomas. We have previously demonstrated the elimination of ESCs by GCV. After 3 treatment cycles, the mice were euthanized in an enclosed chamber filled with carbon dioxide, followed by cervical dislocation, and their tumor tissues and surrounding skin tissues were examined.

### RT-qPCR

We used RNeasy Fibrous Tissue Mini kit (Qiagen) to isolate the RNA from the UVM tissues and surrounding skin tissues following the manufacturer’s instructions, then quantified the total RNA by absorption at 260 nm as previously described [17]. Next, a PrimeScript™ RT Master Mix (Takara, Japan) was used to generate cDNA, which was used for qPCR with SYBR® Premix Ex Taq™ (Takara) in a StepOnePlus thermal cycler (ABI, USA). The GAPDH gene was served as the internal reference.

### Immunohistochemical (IHC) staining

Immunohistochemistry was performed for tumor and skin tissues according to the standard procedure using the following primary antibodies: XRCC6 (ab92450; Abcam), LIG4 (ab193353; Abcam), PARP1 (ab191217; Abcam), DNA-PK (#38168; Cell Signaling) and XRCC5 (WH0007520M2; Sigma-Aldrich). Slides were imaged on a Pannoramic Digital Slide Scanner (3DHISTECH, Hungary) and analyzed by Image-Pro Plus 6.0 (Media Cybemetics, USA). Paraffin sections were deparaffinized and hydrated in PBS. Following blocking of endogenous peroxidase, sections were pretreated with appropriate buffer, if necessary. Thereafter, sections were incubated with primary antibody at 4˚C overnight. Following washing three times with PBS, sections were subsequently incubated with MAX-PO. Peroxidase activity was visualized with diaminobenzidine (DAB).

### Terminal dUTP nick end‐labelling (TUNEL) assay

TUNEL assay was used to detect apoptosis in tumor tissue. It was performed following the manufacturer’s (KeyGen’s) instructions and analyzed by Image-Pro Plus 6.0.

### Statistical analysis

The survival times were compared using Kaplan–Meier analysis, and the *p* value was calculated using the log-rank test. Statistical analysis was performed using GraphPad Prism software. A 2-tailed unpaired Student t-test was used for analyses comparing only 2 groups, and analysis of variance and an appropriate post hoc test were used for analyses comparing more than 2 groups. Statistical significance was set at *p* < 0.05.

## Results

### Correlation between NHEJ pathway-related genes and clinicopathological parameters in patients with UVM

**Fig. 1:**
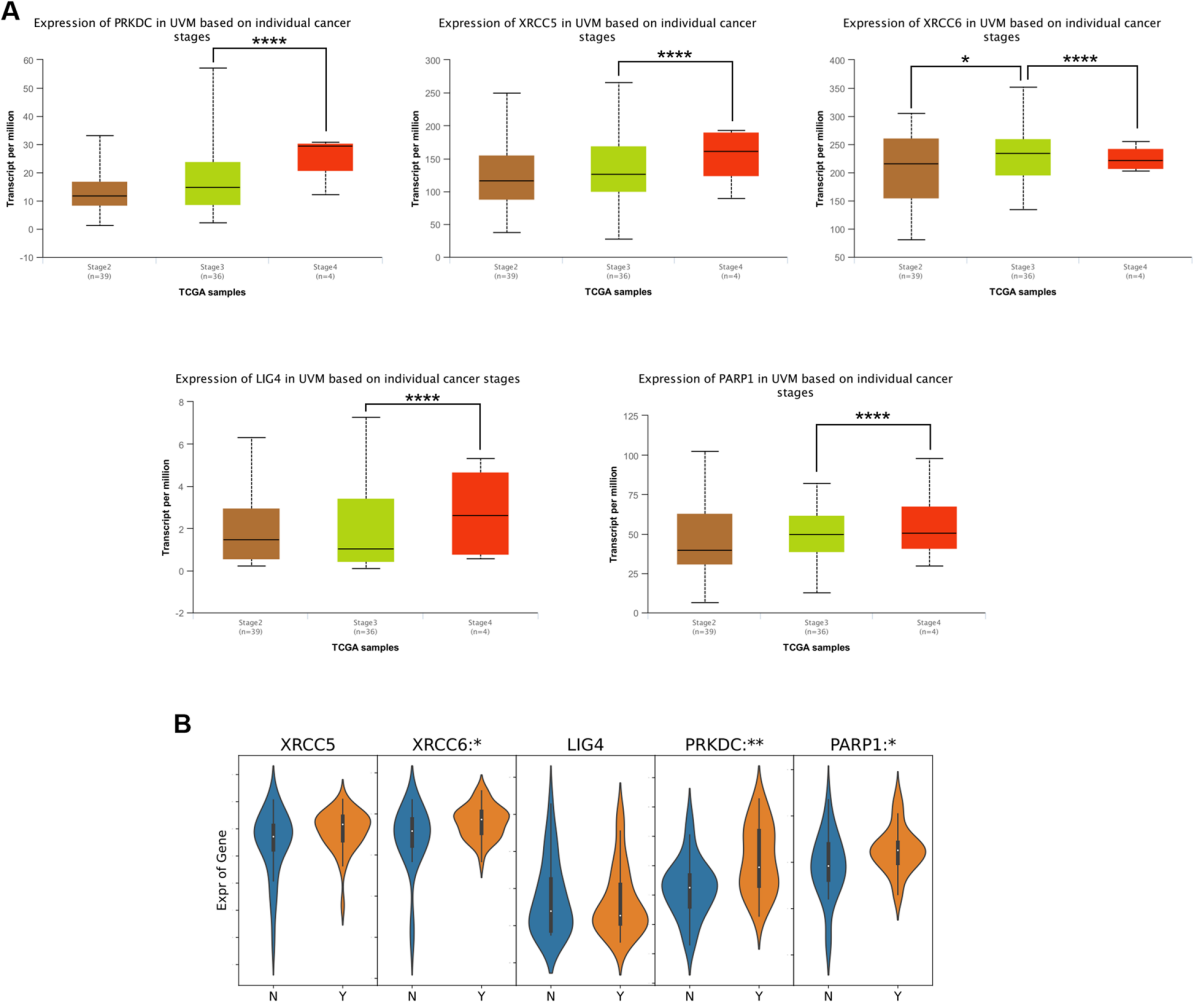
Correlation between NHEJ pathway-related genes and clinicopathological characteristics in patients with UVM. **A** The expression of NHEJ pathway-related genes in different tumor stages of UVM patients (UALCAN). **B** The relative expression level of NHEJ pathway-related genes in UVM patients with metastasis (Y group, *n* = 35) and without metastasis (N group, *n* = 26) (BioStudies database: E-GEOD-22138). The *p* value was set at 0.05. * represents *p* < 0.05, ** represents *p* < 0.01, *** represents *p* < 0.001, **** represents *p* < 0.0001

DDR is a complex network of pathways and proteins, and alterations in any components of the DDR may also contribute to the overall DNA repair deficiency observed in UVM. Since nonhomologous end joining (NHEJ) is the main DNA repair mechanism in UVM [7], we focused on the expression of NHEJ pathway-related genes in UVM subgroups based on individual cancer stages in the data of UALCAN database. As shown in Fig. 1A, for patients with UVM, DNA-PK (alias PRKDC), XRCC6, XRCC5, LIG4 and PARP1 were markedly statistically significant during the transition from stage 3 to stage 4. We also analyzed the relationship of NHEJ pathway-related genes expression and tumor metastasis of UVM patients in a BioStudies database and found that expression of XRCC6, PRKDC and PARP1 was significantly higher in patients with metastasis than in those without metastasis (Fig. 1B). These data strongly suggested that the NHEJ pathway plays a significant role in the UVM progression.

### The prognostic value of DDR related genes in UVM

**Fig. 2:**
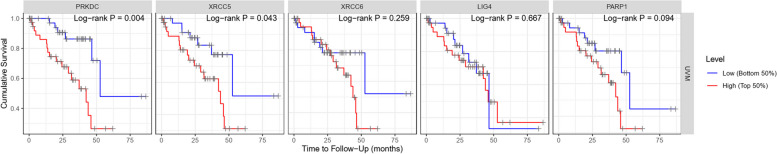
The prognostic value of NHEJ pathway-related genes in UVM patients (80 patients with 23 dying) (TIMER). Patients were divided into high expression group (red, *n* = 40) and low expression group (blue, *n* = 40) according to the median amount of gene expression. The overall survival curves comparing patients with high and low expressions of NHEJ pathway-related genes were plotted. The threshold of *p* value is 0.05

**Fig. 3:**
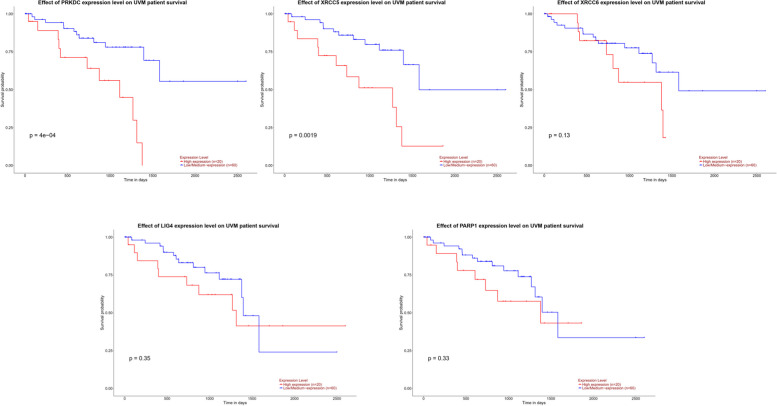
The prognostic value of NHEJ pathway-related genes in UVM patients (UALCAN). The overall survival curves comparing patients with high (red) and low (blue) expression of NHEJ pathway-related genes were plotted. The threshold of *p* value is 0.05

**Fig. 4:**
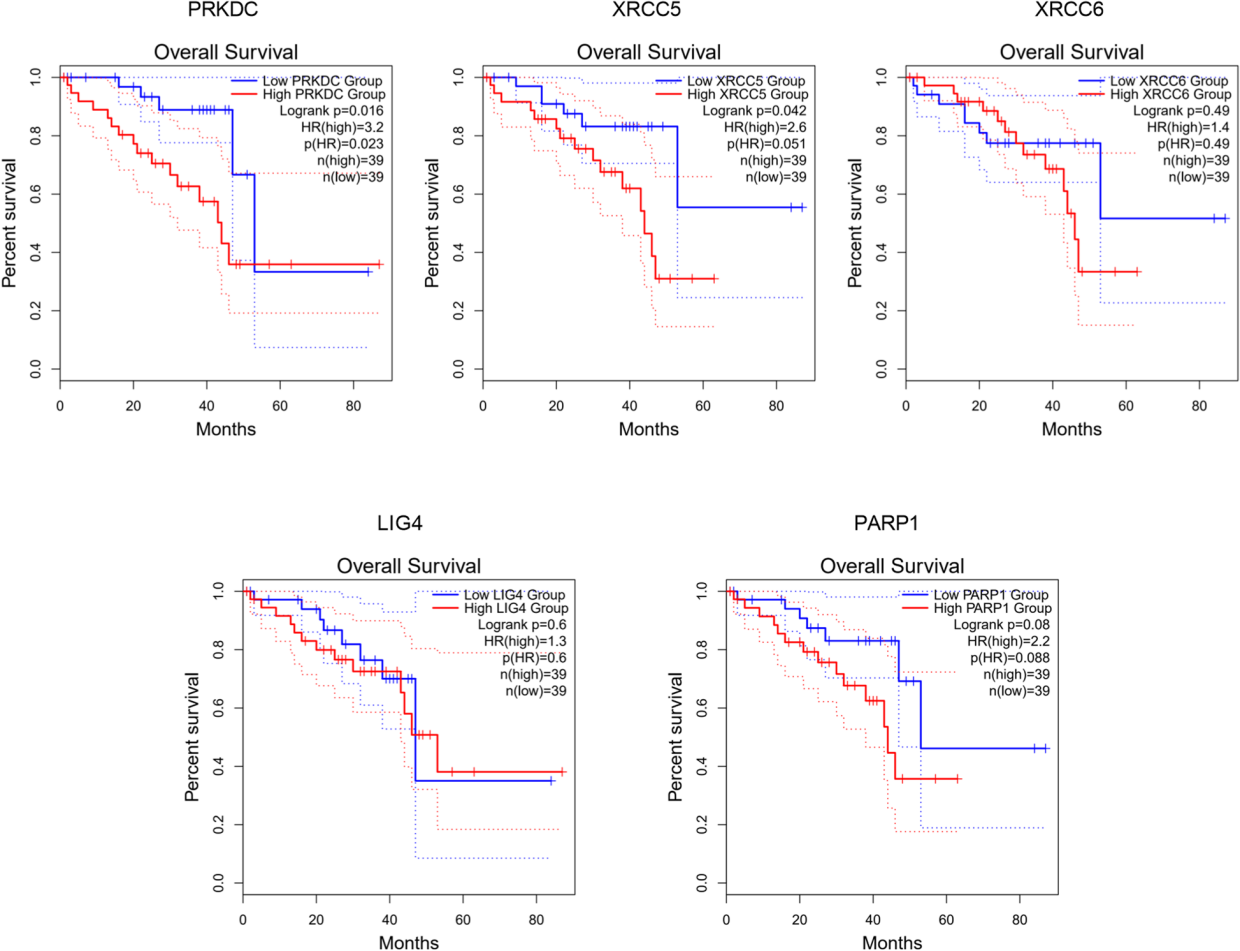
The prognostic value of NHEJ pathway-related genes in UVM patients (GEPIA). The overall survival curves comparing patients with high (red) and low (blue) expression of NHEJ pathway-related genes were plotted. The threshold of *p* value is 0.05

**Fig. 5:**
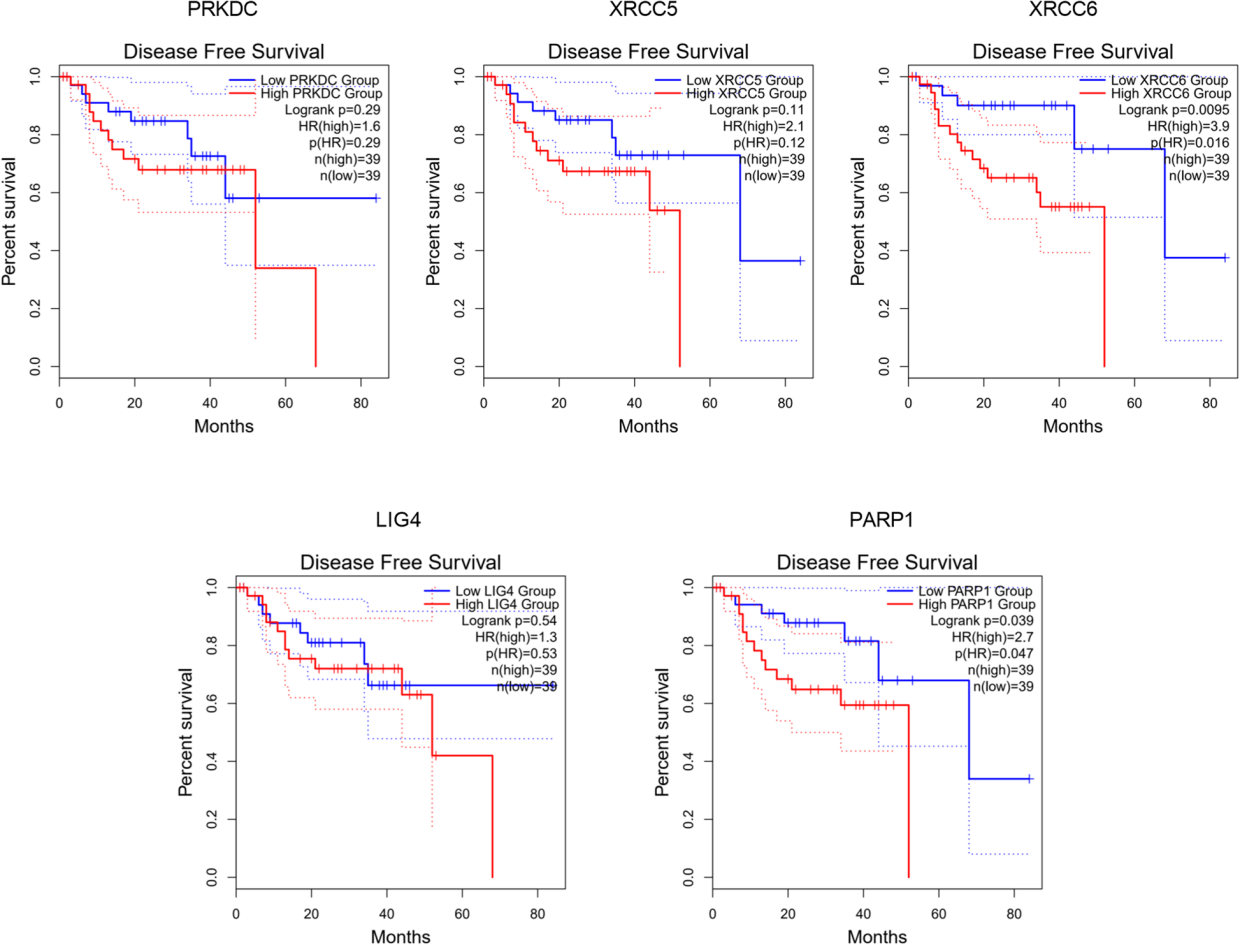
The prognostic value of NHEJ pathway-related genes in UVM patients (GEPIA). The disease-free survival curves comparing patients with high (red) and low (blue) expression of NHEJ pathway-related genes were plotted. The threshold of *p* value is 0.05

We investigated the association of NHEJ pathway-related genes expression and prognosis of UVM patients in TIMER, UALCAN and GEPIA databases. The Kaplan–Meier curve analyses showed that high expression of PRKDC and XRCC5 was markedly associated with lower OS in UVM patients (Figs. 2, 3 and 4). We also used the GEPIA dataset to compare the mRNA expression of the above genes to the DFS of patients with UVM. DFS curves are presented in Fig. 5. The results showed that the UVM patients with low expression of PARP1 and XRCC6 predicted better DFS. Taken together, survival analysis showed that decreased expression of NHEJ pathway-related genes was significantly associated with improved prognosis in UVM patients.

### ESCMe inhibits the NHEJ pathway in UVM cells

**Fig. 6:**
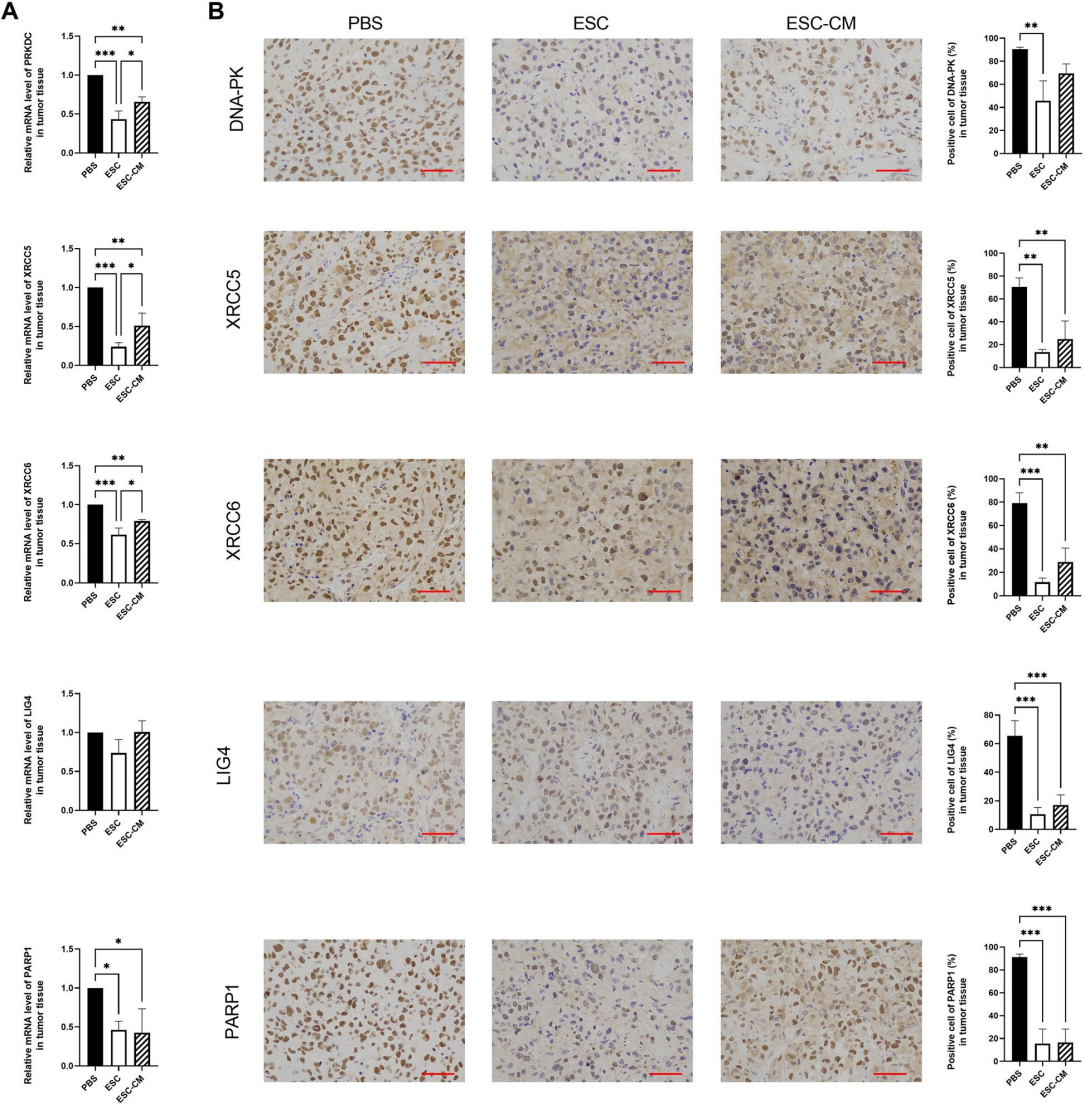
ESCMe inhibits the NHEJ pathway of UVM cells in vivo. **A** Expression of NHEJ pathway-related genes in UVM tissues, as assessed by RT-qPCR. **B** Staining of NHEJ pathway-related factors in UVM tissues obtained from mice 21 days after treatment with PBS, ESCs, or ESC-CM. Data are means ± SDs. *P < 0.05; **P < 0.01; ***P < 0.001; ****P < 0.0001. Scale bar, 50 μm

As reported in our previous article [17], we transplanted ESCs into mice bearing UVM cancer to recapitulate the early embryonic microenvironment and demonstrated that ESCs can reverse the malignant phenotype of C918 cells. Now we performed a quantitative gene expression analysis of the key NHEJ pathway-related genes in UVM tumors. PRKDC, XRCC6, XRCC5 and PARP1 were significantly downregulated in tumor tissue of mice treated with ESCs and ESC-CM compared with those of mice treated with PBS (Fig. 6). Immunohistochemistry analysis revealed that the expression levels of all these NHEJ pathway-related factors were pronouncedly decreased after ESC treatment whereas only the expression levels of XRCC5, XRCC6, LIG4 and PARP1 decreased in the tumors in the ESC-CM treated mice compared with those from the control group. Our results indicate that the expression of nearly all these genes was significantly altered in the ESC treatment group compared with the ESC-CM treatment group, suggesting that the direct cell–cell contact approach with ESCs is more effective than their secreted factors in suppressing the NHEJ pathway. Taken together, these results suggest that the ESCMe significantly inhibited the NHEJ pathway of UVM in vivo.

### ESCMe promotes apoptosis in UVM tissues

**Fig. 7:**
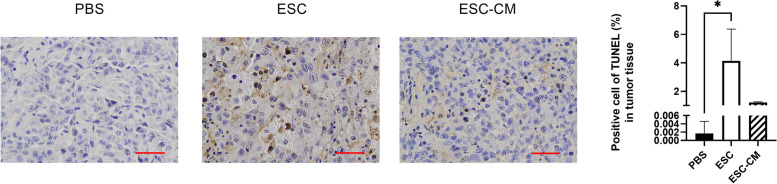
TUNEL staining in UVM tissues. Data are means ± SDs. *P < 0.05. Scale bar, 50 μm

Recent studies have demonstrated that UVM had a reliance for NHEJ in terms of double strand break (DSB) repair and NHEJ is vital to the survival of UVM. If the NHEJ pathway is impaired or overwhelmed, it can lead to persistent DNA damage and activation of apoptotic pathways, resulting in cell death. To determine whether ESCMe treatment affects cell apoptosis in UVM tissues, we performed the TUNEL assay. TUNEL-labeled cells were sporadically positive in the tumor tissue of PBS-treated mice, and the number of positive cells increased only after ESC treatment (Fig. 7). Compared with the control group, the number of apoptotic cells in the ESC-CM treatment group increased, but the difference was not statistically significant. Therefore, ESC treatment can trigger apoptosis in mice bearing UVM cancer, which may be related to the downregulation of the NHEJ pathway in the tumor.

### ESCMe improves the DDR activities of normal cells

**Fig. 8:**
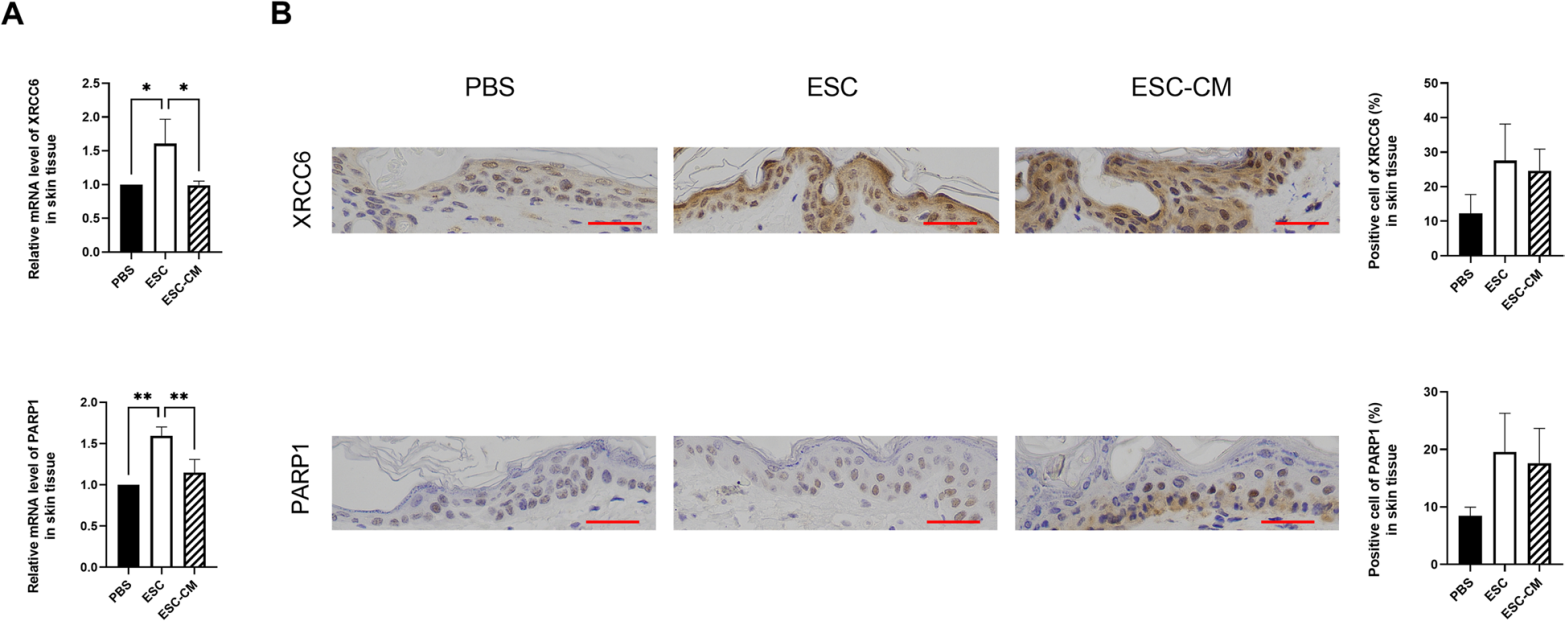
ESCMe improves the DDR activities of normal tissue. **A** Expression of NHEJ pathway-related genes in skin tissues, as assessed by RT-qPCR. **B** Staining of NHEJ pathway-related factors in skin tissues obtained from mice 21 days after treatment with PBS, ESCs, or ESC-CM. Data are means ± SDs. *P < 0.05; **P < 0.01; ***P < 0.001; ****P < 0.0001. Scale bar, 50 μm

Administration of toxic drugs, such as DDR targeted agents and/or high dosage for inducing cell death can cause severe side effects and circumscribe their use as viable therapeutic strategies. To investigate whether ESC treatment impairs the DDR activities of normal cells, we examined the key genes involved in the NHEJ pathway of surrounding normal tissue. Following ESC treatment, while the expressions of NHEJ pathway-related genes in tumors were reduced, whereas the levels of XRCC6 and PARP1 were clearly enhanced in surrounding skin tissue (Fig. 8). The expression levels of XRCC6 and PARP1 showed no obvious change transcriptionally and translationally in ESC-CM treated skin tissue. Combined with our previous research results showing that ESCs could suppress the aggressive phenotype of tumor cells while promoting the proliferation of normal somatic cells, upregulation of DDR activities by ESC treatment could efficiently remove DNA damage from normal cells, contributing to accelerating cell cycle progression and promoting cell proliferation.

## Discussion

UVM is notoriously resistant to both radiation and DNA-damaging agents, which is closely related to its high DDR activities, underlining the importance of targeting the DNA repair capacity as a potentially effective therapeutic option, either alone or as a sensitizer for other treatments [6–8]. The present study showed that a low level of NHEJ pathway-related genes predicted better prognosis in UVM patients and ESCMe could significantly impair the DDR in UVM tissues by downregulating the NHEJ pathway. Previously, we found that the ESCMe suppressed the proliferation, invasiveness, and tumorigenicity of UVM [17]. Combining these results, we can conclude that the ESCMe could inhibit various malignant phenotypes of UVM, indicating a promising and attractive prospect of ESC-based therapy to enhance the sensitivity of UVM to radiochemotherapy and improve treatment outcomes.

DSBs, which can be induced by radiation and other types of genotoxic noxae or during replication are mainly repaired by homologous recombination (HR) and NHEJ [18–20]. Evidence suggests that NHEJ activity was upregulated and was the main mechanism for repairing DNA damage in UVM [7]. The inhibition of the NHEJ protein DNA-PK is even lethal to UVM. In the previous study, we demonstrated that UVM cells treated with ESCs had higher expression levels of p21 [17]. p21 induction is essential for the onset of cell cycle arrest in the DDR, arresting cells at the G1/S transition and giving cells time to repair critical damage [21]. If DNA repair is incomplete, such as when NHEJ-related factors are decreased or DNA damage is extensive, the repair of DSBs can be compromised and the cell undergoes apoptosis [9, 10]. The data presented here, demonstrating the inhibition of functional NHEJ in UVM tissue of ESC-treated mice, was confirmed by gene expression analysis of PCR and immunohistochemical detection. It would therefore be reasonable to consider that the increase in tumor apoptosis by ESCs may be implicated in the upregulation of p21 and downregulation of NHEJ.

Apart from its important role in NHEJ, PARP-1 also engages in other DNA repair mechanisms, such as base excision repair (BER), nucleotide excision repair (NER), DNA mismatch repair (MMR), and maintenance of replication fork stability [22–24], regulating cell death in the event of excessive damage. Several studies have indeed reported that an elevated level of PARP-1 expression was observed in UVM and was associated with a shorter overall survival time and disease-free survival time [6, 8, 25, 26]. It is possible that PARP-1 inhibition can suppress damaged DNA repair and improve tumor killing [27]. Therefore, induced downregulation of PARP-1 by ESCs could lead to multiple compromised DNA repair pathways and a reduced ability to repair DNA damage, suggesting ESCs as an attractive therapeutic option alone or in combination with other treatments of UVM.

Although DDR inhibitors have shown promising results in cancer treatment, they can disrupt the normal DNA repair mechanisms in healthy cells, leading to the accumulation of DNA damage, causing cell cycle arrest or apoptosis, potentially interfering with the ability of normal tissues to regenerate and repair, especially in rapidly dividing tissues such as the bone marrow, skin, and gastrointestinal tract [28–30]. These limit the clinical application of DDR inhibitors [31]. Surprisingly, this study shows that while the expression of NHEJ pathway-related genes in tumors decreased, the levels of XRCC6 and PARP1 were enhanced in the surrounding skin tissue, indicating an upregulation of DDR activities. This enhanced DDR response may efficiently remove DNA damage and maintain genome stability in normal cells, avoiding apoptotic pathways triggered by DNA damage, facilitating faster cell cycle turnover and promoting cell proliferation [32]. This was consistent with our previous research showing that ESCs could markedly suppress tumor growth and enhance the proliferation of adjacent skin tissue [17]. By promoting efficient DNA repair, normal cells with elevated DDR levels are better equipped to cope with various genotoxic stresses, such as radiation or chemical-induced DNA damage. This increased resistance to DNA damage can contribute to the survival and maintenance of normal cells, potentially mitigating concerns about severe side effects of cancer therapy.

It is worth noting that the suppressive effects of the ESCMe on the DDR activities of UVM were much more significant in the ESC treatment group than in the ESC-CM treatment group, which was in agreement with our previous finding that ESC treatment showed a superior therapeutic effect to ESC-CM treatment in terms of proliferation, invasiveness, and tumorigenicity [17]. Direct cell-to-cell contact with ESCs appears to be more effective in inhibiting the NHEJ pathway and triggering apoptosis in tumor cells than exposure to their secreted factors. Furthermore, the secreted factors of ESCs showed no obvious effect on the DDR of normal cells. This may be due to the direct signal communication via cell–cell contact, which is the main mechanism by which ESC influence the DDR activity of both cancer cells and normal cells.

Nevertheless, this study has several limitations. First, this study only illustrated the effect of ESCMe on DDR in subcutaneous UVM models, the conditions of which could be different from that in orthotopic models. Further research is needed to explore the influence of ESCMe on orthotopic UVM models. Second, the experiments in this study were only performed on the C918 cell line. Further studies should be conducted in more UVM cell lines. Third, because of the lack of clinicopathological data on UVM in relevant databases such as TCGA, we were unable to find more data for effective analysis. Clinical data of more UVM patients need to be further collected.

## Conclusions

Our study provides evidence that ESCs can effectively suppress the DDR activity of tumor cells in subcutaneous UVM models while promoting DNA repair in normal cells. These findings capitalize on the unique properties of ESCs in modulating the DDR response in tumors and normal tissues, supporting the potential therapeutic use of ESCs as a viable approach for UVM treatment while minimizing damage to healthy cells. Future studies of ESCs on orthotopic UVM models are needed to evaluate the role of ESCs in ocular environment and the mechanistic association with DDR activities. Such approaches could significantly impact cancer treatment paradigms and provide new avenues for improving patient outcomes in the battle against cancer.

### Supplementary Information

**Supplementary Material 1.**

## Data Availability

All data in the manuscript is available through the responsible corresponding author.

## Abbreviations

ESCs: Embryonic stem cells
PI3K: Phosphoinositide 3-kinase
TK: Thymidine kinase
ESCMe: Embryonic stem cell microenvironment
TUNEL: TdT-mediated dUTP nick-end labeling
RT-qPCR: Reverse transcription polymerase chain reaction
ESC-CM: Embryonic stem cell-conditioned medium
PBS: Phosphate-buffered saline
HSV-TK: Herpes simplex virus thymidine kinase
GCV: Ganciclovir
UVM: Uveal melanoma
DDR: DNA damage response
NHEJ: Nonhomologous end joining
TCGA: The cancer genome atlas
OS: Overall survival
DFS: Disease-free survival
DAB: Diaminobenzidine
DSB: Double strand break
HR: Homologous recombination
BER: Base excision repair
NER: Nucleotide excision repair
MMR: DNA mismatch repair
