# Supplementary material for: Modulating DNA damage response in uveal melanoma through embryonic stem cell microenvironment
Source: BMC Cancer. 2024 Apr 24;24:519. doi: 10.1186/s12885-024-12290-x (PMC11040824; doi:10.1186/s12885-024-12290-x)
Supplement: Supplementary file 1 — Supplementary Material 1. [file 12885_2024_12290_MOESM1_ESM.docx]

**Table S1. Primer sequences**

| **Primer** | **Forward** | **Reverse** |
| --- | --- | --- |
| Human-GAPDH | CGTATTGGGCGCCTGGTCAC | ATGATGACCCTTTTGGCTCC |
| Human-PRKDC | AGGACCGTGCAAGGTTATA | GTGTCTGTCTGGGTGAGGA |
| Human- XRCC6 | GGAGTCGCTGGTGATTGGG | GGATGTTCCTGCGGGGTGT |
| Human- XRCC5 | TGAGATTGCTTTAGTCCTG | ATTTTGCTTTCAATGTCCT |
| Human- LIG4 | GGAGATGCTGGAGACTTTG | GTCGTTTACTTGCTGTATGGT |
| Human- PARP1 | TCTCAAATCGCTTTTACAC | CTGCTATCATCAGACCCTC |
| Mouse- Gapdh | AGTGTTTCCTCGTCCCGTAGA | TGAGGTCAATGAAGGGGTCGT |
| Mouse-Xrcc6 | AAGCCTTTTCCAGTGAGAC | CTGCCGGTGTTTACATTAA |
| Mouse- Parp1 | CTGGTCTTTAAGAGCGACG | TTTCGGCTAGGATTCTGTG |
